# Supplementary material for: Metabolomics of a neonatal cohort from the Alliance for Maternal and Newborn Health Improvement biorepository: Effect of preanalytical variables on reference intervals
Source: PLoS One. 2023 Jan 6;18(1):e0279931. doi: 10.1371/journal.pone.0279931 (PMC9821480; doi:10.1371/journal.pone.0279931)
Supplement: S1 Table — Appropriate for gestational age (AGA) means the baby’s weight is appropriate for the GA (weight between 10th and 90th percentile). Small for gestational age (SGA) means a baby’s weight is less than expected for the GA (weight less than 10th percentile). *p-values are calculated using two sample t-test for gender difference. (DOCX) [file pone.0279931.s001.docx]

**Supplementary Table 1: Gender differences in reference intervals of amino acids, succinylacetone and acylcarnitine concentrations in dried blood spot of neonates born to Pakistani mothers based on gestational age and birth weight.**

| **Grouping** | | **Group I**  **AGA babies**    **(Including preterm and post term babies)** | | | | **Group II**  **Full Term babies**    **(Including AGA + SGA)** | | | | **Group III**  **AGA and Full-Term babies only**    **(Excluding premature, post mature and SGA)** | | | |
| --- | --- | --- | --- | --- | --- | --- | --- | --- | --- | --- | --- | --- | --- |
| Analytes/ Markers in µmol/L; (2.5%–97.5%) | | Overall | Male | Female | *p* value | Overall | Male | Female | *p* value | Overall | Male | Female | *p* value |
|  |  | n=391 | n=181 | n=210 |  | n=515 | n=234 | n=281 |  | n=334 | n=154 | n=180 |  |
| Amino acids | Alanine | 116.41-438.39 | 114.76-480.61 | 113.8-395.93 | 0.8898 | 129.02-462.77 | 126.37-483.06 | 129.36-438.41 | 0.9456 | 128.79-438.91 | 129.37-478.82 | 125.71-408.85 | 0.8401 |
|  | Arginine | 1.57-9.01 | 1.55-9.3 | 1.58-8.93 | 0.587 | 1.57-8.67 | 1.53-9.1 | 1.57-8.49 | 0.539 | 1.56-7.98 | 1.53-9.1 | 1.57-7.97 | 0.5716 |
|  | Arginosuccinic acid | 0.01-0.03 | 0.01-0.03 | 0.01-0.03 | 0.4832 | 0.01-0.03 | 0.01-0.04 | 0.01-0.03 | 0.053 | 0.01-0.03 | 0.01-0.03 | 0.01-0.03 | 0.1687 |
|  | Citrulline | 5.59-18.23 | 5.26-18.09 | 5.61-18.73 | 0.2355 | 5.96-20.1 | 6.08-20.57 | 5.7-18.89 | 0.5799 | 5.62-18.43 | 5.71-17.99 | 5.47-19.42 | 0.4394 |
|  | Glutamine | 162.07-464.89 | 152.81-472.55 | 173.39-468.42 | 0.5987 | 171.84-460.1 | 158.85-466.35 | 180.04-453.05 | 0.7001 | 167.47-461.71 | 154.11-466.35 | 174.64-457.75 | 0.8024 |
|  | Leucine | 91.73-294.64 | 89.89-296.98 | 92.11-293.97 | 0.4865 | 91.6-294.53 | 89.29-298.89 | 93.13-292.52 | 0.5563 | 90.88-295.11 | 89.29-295.93 | 92.45-297.73 | 0.6353 |
|  | Methionine | 11.22-35.63 | 11.12-39.82 | 11.21-31.38 | 0.2388 | 11.35-33.92 | 11.75-41.09 | 11.2-31.72 | 0.1284 | 11.26-32.85 | 11.71-38.25 | 10.66-30.63 | 0.1355 |
|  | Ornithine | 14.07-49.88 | 14.73-49.77 | 13.9-50.59 | 0.4505 | 14.23-53.73 | 14.78-55.63 | 14.15-53.67 | 0.4666 | 14.18-50.05 | 16.11-49.76 | 13.98-50.97 | 0.3547 |
|  | Phenylalanine | 42.07-122.5 | 42.19-117.77 | 41.84-125.37 | 0.8638 | 42.25-124.99 | 41.97-130.29 | 42.99-124.77 | 0.8778 | 42.02-123.41 | 42.25-119.16 | 41.56-126.42 | 0.954 |
|  | Tyrosine | 47.62-209.16 | 46.76-243.32 | 47.87-207.02 | 0.1982 | 47.67-191.66 | 46.85-199.16 | 47.82-181.91 | 0.0602 | 46.72-190.04 | 46.56-237.44 | 46.31-183.58 | 0.1549 |
|  | Valine | 66.12-179.92 | 64.66-177.55 | 67.6-184.79 | 0.8882 | 66.76-191.98 | 64.3-195.65 | 68.7-189.83 | 0.729 | 66.73-184.32 | 64.3-181.55 | 70.7-187.95 | 0.8897 |
| Organic acid | Succinylacetone | 0.5-1.02 | 0.5-1.06 | 0.49-1 | 0.6211 | 0.5-1 | 0.47-1.03 | 0.5-0.98 | 0.7481 | 0.5-0.96 | 0.49-0.97 | 0.5-0.96 | 0.251 |
| Acylcarnitine | C0 | 12.82-47.97 | 14.85-48.2 | 12.32-48.37 | 0.0135 | 12.92-48.32 | 14.6-47.96 | 12.45-50.82 | 0.0011 | 12.46-48.82 | 12.97-50.94 | 12.45-45.72 | 0.0113 |
|  | C2 | 9.46-46.53 | 10.93-48.56 | 9-41.74 | 0.0013 | 9.4-46.31 | 10.34-47.69 | 9.12-42.68 | 0.0029 | 9.9-45.02 | 10.36-48.33 | 9.44-40.1 | 0.0003 |
|  | C3 | 0.72-4.48 | 0.8-4.72 | 0.68-4.32 | 0.1608 | 0.71-4.65 | 0.79-4.79 | 0.68-4.54 | 0.3991 | 0.73-4.52 | 0.76-4.72 | 0.73-4.34 | 0.1708 |
|  | C3-DC | 0.02-0.05 | 0.02-0.06 | 0.02-0.05 | 0.0005 | 0.02-0.05 | 0.02-0.06 | 0.02-0.05 | 0.0002 | 0.02-0.05 | 0.02-0.06 | 0.02-0.05 | 0.0008 |
|  | C4 | 0.1-0.61 | 0.12-0.63 | 0.1-0.62 | 0.5651 | 0.1-0.62 | 0.12-0.61 | 0.1-0.74 | 0.2429 | 0.1-0.68 | 0.1-0.73 | 0.1-0.63 | 0.3356 |
|  | C4-DC | 0.07-0.27 | 0.07-0.27 | 0.07-0.28 | 0.1351 | 0.06-0.27 | 0.06-0.26 | 0.05-0.29 | 0.0177 | 0.07-0.27 | 0.06-0.29 | 0.07-0.27 | 0.1162 |
|  | C4-OH | 0.05-0.32 | 0.06-0.32 | 0.05-0.31 | 0.0019 | 0.05-0.31 | 0.06-0.31 | 0.05-0.31 | 0.0022 | 0.05-0.32 | 0.06-0.33 | 0.05-0.27 | 0.0005 |
|  | C5 | 0.05-0.3 | 0.05-0.3 | 0.05-0.28 | 0.6626 | 0.05-0.3 | 0.06-0.3 | 0.05-0.32 | 0.4432 | 0.05-0.3 | 0.05-0.3 | 0.05-0.3 | 0.9217 |
|  | C5:1 | 0.01-0.02 | 0.01-0.02 | 0.01-0.02 | 0.1614 | 0.01-0.02 | 0.01-0.02 | 0.01-0.02 | 0.0132 | 0.01-0.02 | 0.01-0.02 | 0.01-0.02 | 0.0941 |
|  | C5-DC | 0.01-0.07 | 0.02-0.07 | 0.01-0.07 | 0.0025 | 0.01-0.08 | 0.02-0.08 | 0.01-0.07 | 0.0046 | 0.01-0.07 | 0.02-0.07 | 0.01-0.07 | 0.011 |
|  | C5-OH | 0.05-0.17 | 0.06-0.17 | 0.05-0.17 | 0.0127 | 0.06-0.17 | 0.06-0.17 | 0.05-0.18 | 0.0428 | 0.05-0.17 | 0.06-0.17 | 0.05-0.19 | 0.0043 |
|  | C6 | 0.02-0.11 | 0.02-0.11 | 0.02-0.09 | 0.0016 | 0.02-0.11 | 0.02-0.11 | 0.02-0.1 | 0.0056 | 0.02-0.11 | 0.02-0.11 | 0.02-0.11 | 0.0034 |
|  | C6-DC | 0.01-0.03 | 0.01-0.03 | 0.01-0.03 | 0.0211 | 0.01-0.03 | 0.01-0.03 | 0.01-0.03 | 0.0063 | 0.01-0.03 | 0.01-0.03 | 0.01-0.03 | 0.0259 |
|  | C8 | 0.03-0.13 | 0.03-0.13 | 0.02-0.14 | 0.0006 | 0.03-0.14 | 0.03-0.14 | 0.02-0.15 | 0.0009 | 0.03-0.14 | 0.03-0.14 | 0.02-0.15 | 0.0033 |
|  | C8:1 | 0.02-0.25 | 0.02-0.26 | 0.02-0.23 | 0.0193 | 0.02-0.24 | 0.02-0.25 | 0.02-0.23 | 0.0078 | 0.02-0.25 | 0.02-0.26 | 0.02-0.24 | 0.0262 |
|  | C10 | 0.04-0.19 | 0.04-0.22 | 0.03-0.19 | 0.0087 | 0.04-0.22 | 0.04-0.24 | 0.03-0.19 | 0.0133 | 0.04-0.2 | 0.04-0.22 | 0.03-0.2 | 0.0328 |
|  | C10:1 | 0.02-0.14 | 0.03-0.14 | 0.02-0.12 | <0.001 | 0.02-0.14 | 0.03-0.15 | 0.02-0.12 | 0.0003 | 0.03-0.14 | 0.03-0.15 | 0.02-0.13 | 0.0001 |
|  | C12 | 0.07-0.41 | 0.08-0.42 | 0.06-0.4 | 0.0039 | 0.07-0.42 | 0.08-0.43 | 0.06-0.4 | 0.0042 | 0.08-0.41 | 0.08-0.42 | 0.07-0.41 | 0.0028 |
|  | C12:1 | 0.03-0.27 | 0.03-0.29 | 0.03-0.24 | 0.0202 | 0.03-0.28 | 0.03-0.3 | 0.03-0.26 | 0.0441 | 0.03-0.26 | 0.03-0.27 | 0.03-0.25 | 0.0276 |
|  | C14 | 0.15-0.55 | 0.17-0.58 | 0.15-0.5 | 0.0007 | 0.16-0.55 | 0.18-0.59 | 0.15-0.52 | 0.0001 | 0.15-0.55 | 0.16-0.56 | 0.15-0.53 | 0.0002 |
|  | C14:1 | 0.06-0.32 | 0.07-0.33 | 0.06-0.31 | 0.0007 | 0.06-0.33 | 0.08-0.35 | 0.06-0.32 | 0.0005 | 0.06-0.32 | 0.08-0.33 | 0.06-0.32 | 0.0005 |
|  | C14:2 | 0.01-0.03 | 0.01-0.03 | 0.01-0.03 | <0.001 | 0.01-0.04 | 0.01-0.04 | 0.01-0.04 | <0.001 | 0.01-0.03 | 0.01-0.04 | 0.01-0.03 | 0.0006 |
|  | C14-OH | 0.01-0.04 | 0.01-0.04 | 0.01-0.04 | 0.0002 | 0.01-0.04 | 0.01-0.04 | 0.01-0.04 | 0.0007 | 0.01-0.04 | 0.01-0.04 | 0.01-0.04 | 0.0003 |
|  | C16 | 1.92-7.01 | 2.03-7.48 | 1.85-6.67 | 0.0007 | 1.94-6.94 | 2.08-7.34 | 1.89-6.69 | 0.0044 | 1.92-6.95 | 2-7.47 | 1.91-6.69 | 0.0003 |
|  | C16-OH | 0.01-0.05 | 0.01-0.05 | 0.01-0.04 | 0.0002 | 0.01-0.05 | 0.01-0.05 | 0.01-0.05 | 0.0001 | 0.01-0.05 | 0.01-0.05 | 0.01-0.05 | 0.0001 |
|  | C16:1 | 0.09-0.43 | 0.12-0.44 | 0.09-0.39 | 0.0004 | 0.1-0.43 | 0.12-0.43 | 0.09-0.44 | 0.0038 | 0.1-0.43 | 0.12-0.43 | 0.09-0.43 | 0.0001 |
|  | C16:1-OH | 0.02-0.09 | 0.02-0.1 | 0.02-0.08 | <0.001 | 0.02-0.09 | 0.02-0.1 | 0.02-0.08 | <0.001 | 0.02-0.09 | 0.02-0.1 | 0.02-0.08 | <0.001 |
|  | C18 | 0.55-2.08 | 0.53-2.35 | 0.59-2.01 | 0.0041 | 0.58-2.07 | 0.53-2.26 | 0.6-1.98 | 0.0166 | 0.54-2.07 | 0.52-2.21 | 0.6-2.03 | 0.0037 |
|  | C18-OH | 0.01-0.02 | 0.01-0.03 | 0.01-0.02 | 0.0015 | 0.01-0.02 | 0.01-0.02 | 0.01-0.02 | 0.0011 | 0.01-0.02 | 0.01-0.02 | 0.01-0.03 | 0.0009 |
|  | C18:1 | 0.76-2.58 | 0.8-3.03 | 0.76-2.39 | 0.0001 | 0.76-2.63 | 0.78-3.01 | 0.76-2.4 | 0.0005 | 0.76-2.56 | 0.72-2.82 | 0.76-2.42 | 0.0002 |
|  | C18:1-OH | 0.01-0.03 | 0.01-0.03 | 0.01-0.03 | 0.0001 | 0.01-0.03 | 0.01-0.03 | 0.01-0.03 | 0.0003 | 0.01-0.03 | 0.01-0.03 | 0.01-0.03 | 0.0001 |
|  | C18:2 | 0.06-0.39 | 0.07-0.39 | 0.06-0.38 | 0.0106 | 0.06-0.36 | 0.07-0.38 | 0.06-0.36 | 0.0045 | 0.06-0.36 | 0.07-0.4 | 0.06-0.35 | 0.0581 |

_Appropriate for gestational age (AGA) means the baby's weight is appropriate for the GA (weight between 10th and 90th percentile). Small for gestational age (SGA) means a baby's weight is less than expected for the GA (weight less than 10th percentile). *p-values are calculated using two sample t-test for gender difference._
