# Supplementary material for: Metabolomics of a neonatal cohort from the Alliance for Maternal and Newborn Health Improvement biorepository: Effect of preanalytical variables on reference intervals
Source: PLoS One. 2023 Jan 6;18(1):e0279931. doi: 10.1371/journal.pone.0279931 (PMC9821480; doi:10.1371/journal.pone.0279931)
Supplement: Attachment — Submitted filename: Rebuttal Letter.docx [file pone.0279931.s002.docx]

28^th^ November 2022

Iman Al-Saleh
Academic Editor
PLOS ONE

**Subject: Rebuttal Letter to article number: PONE-D-22-19104, titled “Metabolomics of a neonatal cohort from the Alliance for Maternal and Newborn Health Improvement Biorepository: Effect of preanalytical variables on reference intervals” submitted to PLOS ONE**

Dear Editor,
We have revised the manuscript as per reviewers and editorial comments and suggestions. We have submitted a revised version of the manuscript (marked and unmarked copies) and addressed all comments point by point below.

**RESPONSES TO ALL SUGGESTIONS AND COMMENTS:**

Journal Requirements: When submitting your revision, we need you to address these additional requirements. 1. Please ensure that your manuscript meets PLOS ONE's style requirements, including those for file naming. The PLOS ONE style templates can be found at 
<https://journals.plos.org/plosone/s/file?id=wjVg/PLOSOne_formatting_sample_main_body.pdf> and 
<https://journals.plos.org/plosone/s/file?id=ba62/PLOSOne_formatting_sample_title_authors_affiliations.pdf>.

**Response:** We have ensured that the manuscript meets PLOS ONE's style requirements and have formatted it accordingly.

2. Please amend your current ethics statement to address the following concerns:
a) Did participants provide their written or verbal informed consent to participate in this study?
b) If consent was verbal, please explain i) why written consent was not obtained, ii) how you documented participant consent, and iii) whether the ethics committees/IRB approved this consent procedure.

**Response:** The consent taken was written and we have added this in the manuscript “Written informed consent was taken before sample collection from parents/ guardians of the newborns and ethical review committee approval was sought.”

3. Please include your full ethics statement in the ‘Methods’ section of your manuscript file. In your statement, please include the full name of the IRB or ethics committee who approved or waived your study, as well as whether you obtained informed written or verbal consent. If consent was waived for your study, please include this information in your statement as well.

**Response:** Full ethics statement has been added in the ‘Methods’ section of the manuscript file. It includes the full name of the IRB or ethics committees who approved the study, and that written informed consent was taken.

4. In your Data Availability statement, you have not specified where the minimal data set underlying the results described in your manuscript can be found. PLOS defines a study's minimal data set as the underlying data used to reach the conclusions drawn in the manuscript and any additional data required to replicate the reported study findings in their entirety. All PLOS journals require that the minimal data set be made fully available. For more information about our data policy, please see <http://journals.plos.org/plosone/s/data-availability>. "Upon re-submitting your revised manuscript, please upload your study’s minimal underlying data set as either Supporting Information files or to a stable, public repository and include the relevant URLs, DOIs, or accession numbers within your revised cover letter. For a list of acceptable repositories, please see <http://journals.plos.org/plosone/s/data-availability#loc-recommended-repositories>. Any potentially identifying patient information must be fully anonymized. Important: If there are ethical or legal restrictions to sharing your data publicly, please explain these restrictions in detail. Please see our guidelines for more information on what we consider unacceptable restrictions to publicly sharing data: <http://journals.plos.org/plosone/s/data-availability#loc-unacceptable-data-access-restrictions>. Note that it is not acceptable for the authors to be the sole named individuals responsible for ensuring data access. We will update your Data Availability statement to reflect the information you provide in your cover letter.

**Response:** The datasets generated and/or analysed during the current study are now available as coded data without any identifiers on FigShare; repository link: <https://figshare.com/articles/journal_contribution/AAACARN_data_for_registry_xlsx/21081391>

5. Your ethics statement should only appear in the Methods section of your manuscript. If your ethics statement is written in any section besides the Methods, please delete it from any other section.

**Response:** The ethics statement is added in the Methods section of the manuscript only.

6. Please review your reference list to ensure that it is complete and correct. If you have cited papers that have been retracted, please include the rationale for doing so in the manuscript text, or remove these references and replace them with relevant current references. Any changes to the reference list should be mentioned in the rebuttal letter that accompanies your revised manuscript. If you need to cite a retracted article, indicate the article’s retracted status in the References list and also include a citation and full reference for the retraction notice.
**Response:** All references have been verified and there is no change in the list.

**Reviewers' comments:**
Reviewer's Responses to Questions

**Comments to the Author**
1. Is the manuscript technically sound, and do the data support the conclusions?
The manuscript must describe a technically sound piece of scientific research with data that supports the conclusions. Experiments must have been conducted rigorously, with appropriate controls, replication, and sample sizes. The conclusions must be drawn appropriately based on the data presented.

Reviewer #1: Partly; Reviewer #2: Yes; Reviewer #3: Yes

**Response to Reviewers’ comments**: not required

2. Has the statistical analysis been performed appropriately and rigorously?

Reviewer #1: Yes; Reviewer #2: Yes; Reviewer #3: Yes

**Response to Reviewers’ comments**: not required

3. Have the authors made all data underlying the findings in their manuscript fully available?
The [PLOS Data policy](https://eur03.safelinks.protection.outlook.com/?url=http%3A%2F%2Fwww.plosone.org%2Fstatic%2Fpolicies.action%23sharing&data=05%7C01%7Clena.jafri%40aku.edu%7C665fbd35e7ec4c55453408da7e7eada0%7Ca5d4252a02f94e6096f09733baae4919%7C0%7C0%7C637961380690002983%7CUnknown%7CTWFpbGZsb3d8eyJWIjoiMC4wLjAwMDAiLCJQIjoiV2luMzIiLCJBTiI6Ik1haWwiLCJXVCI6Mn0%3D%7C3000%7C%7C%7C&sdata=Vjy3HxQOus3%2BgdgKGtBIpp21wFeMk4IwZJwmd7cC2mI%3D&reserved=0) requires authors to make all data underlying the findings described in their manuscript fully available without restriction, with rare exception (please refer to the Data Availability Statement in the manuscript PDF file). The data should be provided as part of the manuscript or its supporting information, or deposited to a public repository. For example, in addition to summary statistics, the data points behind means, medians and variance measures should be available. If there are restrictions on publicly sharing data—e.g. participant privacy or use of data from a third party—those must be specified.

Reviewer #1: Yes; Reviewer #2: No; Reviewer #3: No

**Response to Reviewers’ comments**: data has been submitted to public repository now. And this information has been updated in the manuscript. The datasets generated and/or analysed during the current study are now available as coded data without any identifiers on FigShare; repository identity number 21081391.

4. Is the manuscript presented in an intelligible fashion and written in standard English?
PLOS ONE does not copyedit accepted manuscripts, so the language in submitted articles must be clear, correct, and unambiguous. Any typographical or grammatical errors should be corrected at revision, so please note any specific errors here.

Reviewer #1: Yes; Reviewer #2: Yes; Reviewer #3: Yes

**Response to Reviewers’ comments**: not required

5. Review Comments to the Author
Please use the space provided to explain your answers to the questions above. You may also include additional comments for the author, including concerns about dual publication, research ethics, or publication ethics. (Please upload your review as an attachment if it exceeds 20,000 characters)

**Reviewer** #**1**: The objective of this study was to determine reference intervals (RI) for dried blood spot measurements of amino acids, acylcarnitines, and succinylacetone of neonates in Pakistan. The authors examined whether mean levels of the analytes differed statistically by gender, term, or gestational age. Although some statistically significant differences were observed, the authors concluded that none of these were clinically significant and, thus, did not recommend separate RI for different subgroups. These are important data to support development of a national newborn screening program.
Comments: Line 150: “171 (53%) were from females”. According to Table 1, 325 samples were from females.

**Response to Reviewers’ comments**: This was a typo error in the results text Line 150 and has been corrected in track changes to “Out of the total 610 DBS, 325 (53.2%) were from females”.

**Reviewer** #**1**: Lines 153-155: “145 (23.7%) neonates being low birth weight (LBW), 141 (23.1%) were SGA and 87 (14.3%) being preterm.” The percentages for normal weight and low birth weight in table 1 appear to be incorrect (e.g., 465/610 is 76.2%, not 75.5%; 145/610 = 23.8%, not 24.5%). Please define normal weight and low birth weight in Methods. Please add data for SGA and AGA to Table 1.

**Response to Reviewers’ comments**: The normal weight and low birth weight have been defined in Methods. Additionally, data for SGA and AGA has been added to Table 1 in the last rows.

**Reviewer** #**1**: When describing the table 2 results, the authors state that although there are statistically significant differences in some of the analytes between males and females, the differences did not seem clinically significant and thus did not justify creation of separate RI. The process for weighing clinical vs. statistical significance is not clear for values with more than negligible differences (e.g., the 97.5% limit for C2 is 47.08 in males vs. 41.48 in females). Perhaps the authors could add to Methods the process for making these judgements. For example, were all statistically significant differences reviewed by one author or several authors who came to a consensus about clinical significance? Was literature consulted?

**Response to Reviewers’ comments**: The methodology of deciding the clinical relevance of the bias that was carried out during the study has been added in the manuscript: “The authors agreed that the statistical significance indicates the reliability of the study results while the clinical significance reflects its impact on clinical practice. Based on this the amino acids and acylcarnitine with statistically significant differences were individually analyzed by three subject experts. The subject experts based on their experiences and literature review noted their decision. This was followed by developing consensus in a meeting regarding the bias (p value) being clinically significant (even if any one expert disagreed) or insignificant (if all agreed).”

**Reviewer** #**1**: Some authors have proposed more objective criteria to determine the need for partitioning (e.g., PMID 11805016). Did the authors consider a similar approach and decide against it? Perhaps the authors could note in Methods or the Discussion why they did not use such an approach.

**Response to Reviewers’ comments**: In this paper we did not find clinically significant difference in any analytes, hence RI partitioning was not done.

**Reviewer** #**1**: Consider expanding table 2 to show the mean and median for males and females separately.

**Response to Reviewers’ comments**: We have expanded table 2 which now includes mean and median values.

**Reviewer** #**1**: Figure 1 footnote: “No statistically significant difference (p value >0.05) was found amongst gender in the amino acids, succinylacetone and acylcarnitine concentrations studied.” This is not accurate as some statistically significant differences were observed for acylcarnitines according to table 2.

**Response to Reviewers’ comments**: : “No statistically significant difference (p value >0.05) was found amongst gender in the amino acids, succinylacetone and acylcarnitine concentrations studied.” This line has been removed from the figure 1

**Reviewer** #**1**: In Line 154 the authors wrote that 141 were SGA, but table 3 shows 219 are SGA. Please clarify.

**Response to Reviewers’ comments**: The total SGA were 219. It has been corrected in the manuscript.

**Reviewer** #**1**: In table 3, testing differences in means does not seem to be the correct focus for establishing RI. A mean value can be statistically significantly different between groups while the RI are very similar, as was shown in table 2.

**Response to Reviewers’ comments**: The table 3 shows the mean difference in all analytes in SGA and term categories. Authors agree that mean is not the correct measure to evaluate a parameter for the RI-partitioning. The discussion has been modified to accommodate the reviewer’s suggestion

**Reviewer** #1: In the footnote of table 3, the authors mention accounting for false discovery rate. Details about this approach and when it was applied should be added to Methods.

**Response to Reviewers’ comments**: There were error in footnotes and we have corrected it in track changes.

**Reviewer** #**1**: For the analyses in figure 2, please report the number of samples collected on each day (i.e., the n for each group). Also, why is day 0 shown in 2a, but not 2b? Also, please clarify in the figure that the x-axis is showing days.

**Response to Reviewers’ comments**: The number of samples collected on each day have been added in the figure. Both Figure 2 has been reconstructed as day 0 was an error and have been resubmitted. Now we have added in the figure that the x-axis is showing days. The figure has been uploaded after checking through the Preflight Analysis and Conversion Engine (PACE) digital diagnostic tool for PLOS requirements.

**Reviewer** #**1**: For the Dietzen study, I am curious if they also stored DBS cards immediately at -80 degrees and if they used a similar assay to measure the metabolites. From lines 276-278, it is not clear if the authors are saying the Dietzen study did not report these details at all in their study or if Dietzen used different methods than theirs so the results cannot be compared? If they did report their methodology, this would be useful to describe as that might suggest that further work needs to be done to determine the impact of storage conditions (e.g., time between collection and freezing, freezer temperature) or other pre-analytic variables on metabolite levels.

**Response to Reviewers’ comments**: Yes the DBS were stored at minus 80 degrees and this has been added in the manuscript in the methods section (line 290). The instrument -principle was same as ours but the details of assay method is not there. Lines 303-305 are for all the studies we have discussed: “Additionally, the differences between the previous studies and our work could be due to differences in methodology, equipment, reference sample, measurement conditions, or from dietary patterns which were not studied or compared.” Lines 296-298. We have tried to bring clarity in the discussion now and have tried to incorporate your suggestions.

**Reviewer** #**2**: Jafri et al. present a relevant and sound research article establishing reference intervals of diverse metabolites for newborn screening in Pakistan. Scientific and statistical methods applied are sound and the study cohort is well described. Findings support conclusions and the manuscript is well written. I only have a minor comment, for Tables 1 and 2 please add a description of what p-values identify (which test statistic was used, which differences were measured) to improve readability.

**Response to Reviewers’ comments**: In both Tables 1 and 2 we have added a description of what p-values identify, the statistic tests used, and which differences were measured.

**Reviewer #**3: This article describes the results metabolite analysis of dried bloodspots in 610 neonates. DBS analysis is critical to modern neonatology and is now mandatory in the U.S. and many other countries. However, it has been shown that these assays are sensitive to environmental factors. This work, therefore, is of critical importance in establishing an appropriate baseline for deployment of DBS metabolite screening globally. The study is designed and executed well, and the article is easy to follow.

My comments for improving the manuscript are provided below:
1) The authors have demonstrated that the metabolites are not associated with GA, weight, gender, and time of sampling. However, it may be the case that a multivariable model can combine multiple weak associations to identify a signature strong associated with these factors. Adding a simple multivariable linear model to the analysis will be very valuable.
**Response to Reviewers’ comments**: The objective of this paper was to determine the RI for amino acids (eleven amino acids and seven ratios), acylcarnitines (thirty-two acylcarnitines and fourteen ratios) and succinylacetone on DBS. Hence the multivariable linear model statistics were not done. We have another paper in writing with separate objectives (on determinants of low birth weight and association of biomarkers) and will run the regression in that.

2) It has been shown that hypertensive disorders of pregnancy and diabetes can have a profound affect on metabolites measured in DBS. It would be great if the authors could add hypertension, preeclampsia, and diabetes to the analysis (if enough patients with these phenotypes are available).

**Response to Reviewers’ comments**: The samples were taken from newborns hence none of them had documented hypertension, preeclampsia, and diabetes.

3) It is unclear to me why this data cannot be shared. PHI information, of course, would require institutional review and approval. But metabolites themselves and associated demographic/phenotypical information is not PHI and is regularly shared publicly. This will increase the impact of the article as others will be able to reuse the data. This is, of course, subject to institutional and governmental regulations in various countries that I may not be aware of.

**Response to Reviewers’ comments**: Datasets generated and/or analysed during the current study are now available as coded data without any identifiers on FigShare; repository identity number 21081391.

Taken together, I believe this is an important article and all my comments can be addressed in a straight-forward revision. I would, therefore, encourage the editorial board to strongly consider the publication of this article.
Reviewed by Nima Aghaeepour
(Voluntary disclosure of my name)

**Response to Reviewers’ comments**: not required

6. PLOS authors have the option to publish the peer review history of their article ([what does this mean?](https://eur03.safelinks.protection.outlook.com/?url=https%3A%2F%2Fjournals.plos.org%2Fplosone%2Fs%2Feditorial-and-peer-review-process%23loc-peer-review-history&data=05%7C01%7Clena.jafri%40aku.edu%7C665fbd35e7ec4c55453408da7e7eada0%7Ca5d4252a02f94e6096f09733baae4919%7C0%7C0%7C637961380690002983%7CUnknown%7CTWFpbGZsb3d8eyJWIjoiMC4wLjAwMDAiLCJQIjoiV2luMzIiLCJBTiI6Ik1haWwiLCJXVCI6Mn0%3D%7C3000%7C%7C%7C&sdata=zwA5g59wtJ4%2FCnBTRSYA8eX3lWXMveqDFHT7Z7wN7gc%3D&reserved=0)). If published, this will include your full peer review and any attached files.
If you choose “no”, your identity will remain anonymous but your review may still be made public.
Do you want your identity to be public for this peer review? For information about this choice, including consent withdrawal, please see our [Privacy Policy](https://eur03.safelinks.protection.outlook.com/?url=https%3A%2F%2Fwww.plos.org%2Fprivacy-policy&data=05%7C01%7Clena.jafri%40aku.edu%7C665fbd35e7ec4c55453408da7e7eada0%7Ca5d4252a02f94e6096f09733baae4919%7C0%7C0%7C637961380690002983%7CUnknown%7CTWFpbGZsb3d8eyJWIjoiMC4wLjAwMDAiLCJQIjoiV2luMzIiLCJBTiI6Ik1haWwiLCJXVCI6Mn0%3D%7C3000%7C%7C%7C&sdata=8EWusLumcuVIdUaad6F5KLUt5UdZUdk%2Bz3qdjTDpLzo%3D&reserved=0).

Reviewer #1: No; Reviewer #2: No; Reviewer #3: No

**Response to Reviewers’ comments**: not required

Regards,

Corresponding Author:

**Dr Lena Jafri**

Associate Professor and Section Head

Chemical Pathology

Department of Pathology and Laboratory Medicine,

The Aga Khan University, Pakistan
